# Giant mucinous cystic neoplasm of the liver in a young asymptomatic woman: A case report

**DOI:** 10.1097/MD.0000000000048635

**Published:** 2026-05-01

**Authors:** Hao-nan Cheng, Ya-kang Jia, Meng-wei Cai, Jun-yan Xu, Wei Guo, Jin Wang

**Affiliations:** a The Third Division, Department of General Surgery, Jiaozuo People’s Hospital, Affiliated Hospital of Henan Medical University, Jiaozuo, Henan, China.

**Keywords:** CA19-9, hepatectomy, liver cyst, liver tumor, mucinous cystic neoplasm

## Abstract

**Rationale:**

Mucinous cystic neoplasm of the liver (MCN-L) is an uncommon cystic tumor with low malignant potential that mainly occurs in women. Because many patients have few or no symptoms and imaging findings may look similar to simple liver cysts or other cystic lesions, it is easy to make an incorrect diagnosis before surgery.

**Patient concerns:**

A 36-year-old woman had no discomfort. A large cystic liver lesion was found by chance during a routine health checkup. She did not report abdominal pain, distension, fever, jaundice, or weight loss.

**Diagnoses:**

Imaging examinations showed a giant multilocular cystic mass about 20 cm in size in segment IV of the liver, with septal enhancement. Serum carbohydrate antigen 19-9 (CA19-9) was markedly increased (553 IU/mL), while other tests were unremarkable. MCN-L was suspected. Histopathology of the resected specimen confirmed a low-grade MCN-L with ovarian-type stroma, and immunohistochemistry showed estrogen and progesterone receptor positivity in the stromal cells.

**Interventions:**

Because the tumor was very large and located close to major hepatic vessels, the patient underwent open segment IV hepatectomy. The whole cystic lesion was removed en bloc without rupture.

**Outcomes:**

The postoperative recovery was smooth, without bleeding, bile leakage, or infection. During 24 months of follow-up, abdominal computed tomography and serum CA19-9 were checked every 6 months. No tumor recurrence or metastasis was observed, and CA19-9 returned to the normal range. The patient’s quality of life remained good.

**Lessons:**

When a very large hepatic cystic lesion is identified in a young woman, especially with elevated CA19-9 and septal enhancement on magnetic resonance imaging, MCN-L should be considered in the differential diagnosis. Because preoperative diagnosis can be difficult, careful assessment of lesion size, internal architecture, and its relationship to major vessels is essential. Complete surgical resection with negative margins remains the preferred treatment, as it reduces the risks of recurrence and malignant transformation.

## 1. Introduction

Mucinous cystic neoplasm of the liver (MCN-L) is a rare cystic tumor with low malignant potential that mainly affects women and is usually located in the left lobe or near the hepatic hilum. Because the tumor grows slowly, many patients have no obvious symptoms for a long time. When the lesion becomes large or compresses surrounding structures, patients may develop upper abdominal discomfort, abdominal distension, or a palpable mass.

In daily clinical work, MCN-L is often misdiagnosed as a simple liver cyst, liver abscess, or other cystic disease. Imaging may show cystic lesions with internal septa or mural nodules, but these findings are not specific. A definite diagnosis usually depends on histopathology and immunohistochemistry.

Here, we report a young woman with a giant MCN-L who presented without obvious symptoms. We describe the clinical course, imaging features, surgical management, and follow-up of this case. More importantly, this case highlights two practical issues in daily work: first, MCN-L may be overlooked when the patient is asymptomatic and the lesion appears cystic on imaging; second, the choice of surgical approach should be made after careful evaluation of tumor size, location, and proximity to major vascular structures.

## 2. Ethics statement

This case report was conducted in accordance with the Declaration of Helsinki. Ethical review was waived by the institutional review board of Jiaozuo People’s Hospital affiliated to Henan Medical University because this study was a single case report and did not involve an interventional research protocol. Written informed consent for publication of the clinical information and images was obtained from the patient.

## 3. Case presentation

### 3.1. Chief complaints

A hepatic space-occupying lesion was detected during a routine physical examination 2 weeks before admission.

### 3.2. History of present illness

A 36-year-old woman was admitted to our hospital because an abdominal ultrasound performed during a health checkup about half a month earlier showed a liver space-occupying lesion. She did not complain of abdominal pain, abdominal distension, nausea, vomiting, fever, jaundice, or weight loss. There was no recent history of trauma or surgery. She came to our department for further diagnosis and treatment.

### 3.3. History of past illness

The patient had no history of hypertension, diabetes, coronary heart disease, viral hepatitis, or tuberculosis. She underwent an appendectomy at 14 years of age. There was no history of other major operations or serious systemic diseases.

### 3.4. Personal and family history

The patient did not drink alcohol or smoke. She denied long-term exposure to toxic substances and long-term hormone use. There was no family history of liver disease, malignant tumors, or infectious diseases.

### 3.5. Clinical findings

On admission, the patient was in good general condition. Her vital signs were stable. The skin and sclera were not icteric. The lungs and heart were normal on auscultation. The abdomen was soft and flat, with no obvious tenderness, rebound pain, or muscle tension. No hepatosplenomegaly or palpable abdominal mass was detected, and bowel sounds were normal. The rest of the physical examination was within normal limits.

### 3.6. Timeline

#### 3.6.1. Half a month before admission

Routine health check revealed a hepatic cystic lesion on ultrasound.

#### 3.6.2. Admission day

Further evaluation in our hospital; laboratory tests and imaging examinations were performed.

#### 3.6.3. Within 1 week after admission

Multidisciplinary discussion was held, and open segment IV hepatectomy was planned.

#### 3.6.4. Surgery day

Open hepatectomy was completed, and the cystic mass was removed en bloc.

#### 3.6.5. Postoperative days 1 to 7

The patient had an uneventful recovery. The drainage tube was removed after the drainage volume decreased and no bile was observed.

#### 3.6.6. Postoperative month 1

The patient returned for review and had good recovery.

#### 3.6.7. Every 6 months thereafter

Follow-up with abdominal computed tomography (CT) and serum CA19-9.

#### 3.6.8. Postoperative month 24

No recurrence or metastasis; CA19-9 level was normal and the patient felt well.

## 4. Diagnostic assessment

### 4.1. Laboratory examinations

Routine blood tests, coagulation function, liver and kidney function, electrolytes, and inflammatory markers were all within normal ranges. Tumor markers such as alpha-fetoprotein and carcinoembryonic antigen were not significantly elevated. Serum CA19-9 was markedly increased (553 IU/mL). Overall, there were no obvious contraindications to surgical resection.

### 4.2. Imaging examinations

Abdominal ultrasound showed a single large cystic lesion in the liver, about 19 to 20 cm in size, with a clear boundary. The internal echo was mainly anechoic with some septa, and there was no obvious blood flow signal in the cystic cavity. The initial impression was a hepatic cystic lesion.

**Figure 1.**
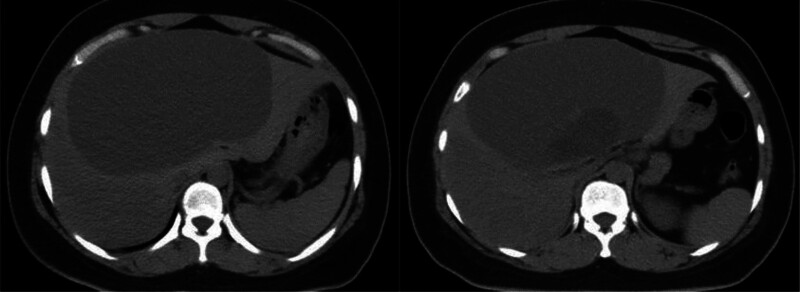
Abdominal CT showing a large multilocular cystic mass with septal enhancement in liver segment IV. CT = computed tomography.

Contrast-enhanced CT (Fig. 1) revealed a large, well-defined cystic mass in liver segment IV, compressing the surrounding hepatic parenchyma. The lesion was mainly low density with visible internal septations. After contrast administration, the septa and cyst wall were mildly enhanced, and no solid component was observed. The lesion was considered a cystic hepatic tumor.

**Figure 2.**
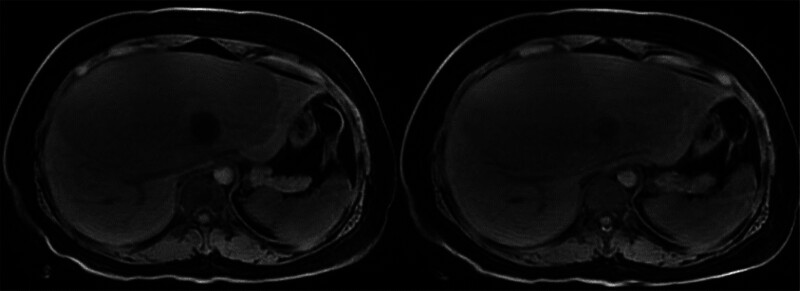
The tumor was located in liver segment IV.

Magnetic resonance imaging (MRI) (Fig. 2) demonstrated a giant multilocular cystic lesion in segment IV. The lesion showed low signal intensity on T1-weighted images and high signal intensity on T2-weighted images. Thin internal septa and the cyst wall were clearly displayed. After contrast enhancement, the septa and cyst wall were enhanced, especially in the arterial and portal venous phases. These findings were in line with a large cystic liver tumor, and MCN-L could not be ruled out.

## 5. Diagnostic reasoning and differential diagnosis

Based on the clinical, laboratory, and imaging findings, a large cystic lesion in liver segment IV was diagnosed. The extremely large size, elevated CA19-9, multilocular structure, and septal enhancement suggested MCN-L. However, we still considered other cystic lesions in the differential diagnosis, such as simple liver cyst and hydatid disease, and planned to confirm the diagnosis by postoperative pathology.

We did not encounter obvious financial, language, or cultural barriers in this case.

## 6. Therapeutic intervention

After multidisciplinary discussion and full communication with the patient and her family, we decided to perform surgical resection. Because the tumor was very large, occupied most of segment IV, and was close to important vessels, we believed that laparoscopic resection would be technically difficult and might increase the risk of intraoperative rupture. Therefore, open segment IV hepatectomy was chosen.

Under general anesthesia, we made an upper abdominal incision. Intraoperatively, a huge cystic mass was found in segment IV of the liver, with a smooth surface and clear boundary. The tumor was close to the middle hepatic vein and left hepatic vein, and it compressed the surrounding liver tissue. There was no obvious invasion of major vessels or bile ducts. No ascites or peritoneal implantation was observed.

We carefully separated the adhesions between the tumor and surrounding tissues, exposed the hepatic hilum, and performed inflow control when necessary. The liver parenchyma was transected along the planned resection line using an energy device and ligation of vessels. The entire cystic lesion was removed en bloc, and there was no rupture of the cyst wall. Hemostasis was secured, and a drainage tube was placed near the resection surface. The specimen was sent for pathological examination.

## 7. Pathological examination

**Figure 3.**
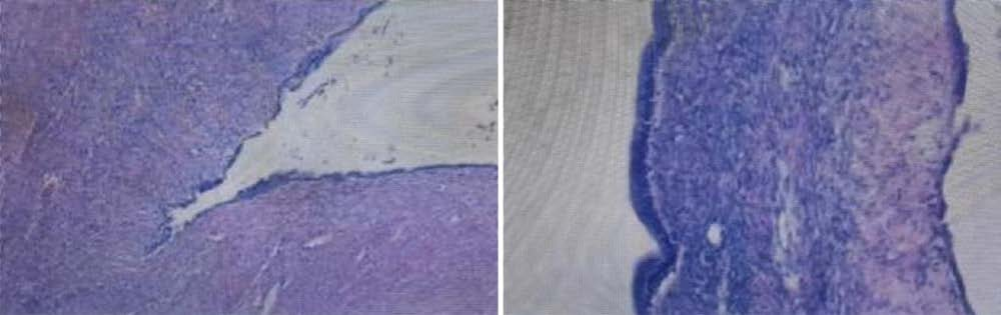
Histopathology (H&E stain, × 200) demonstrating ovarian-type stroma and mucinous epithelium. H&E = hematoxylin and eosin.

Macroscopically, the specimen (Fig. 3) was a multilocular cystic mass measuring approximately 20 × 12 × 6 cm. In some areas the cyst wall was thickened, and most of the inner surface was smooth. Some cavities contained mucinous fluid. No obvious solid nodule was seen.

Microscopically, the cyst wall was lined by a single layer of cuboidal or columnar mucinous epithelium. Beneath the epithelium there was dense ovarian-type stroma composed of spindle-shaped cells. No obvious severe atypia or invasive growth was found. Immunohistochemistry revealed that the stromal cells were positive for estrogen receptor and progesterone receptor, which supported the diagnosis of MCN-L.

## 8. Follow-up and outcomes

The operation went smoothly, and the patient was transferred to the ward in stable condition. Postoperatively, she received routine antibiotics, liver-protective therapy, and fluid supplementation. There was no obvious bleeding, bile leakage, or infection. The drainage tube was removed after the drainage volume decreased and no bile was observed.

The patient was discharged in good condition. She was followed up regularly in the outpatient clinic. Abdominal CT was performed every 6 months, and serum CA19-9 was rechecked. At 24 months after surgery, there was no evidence of tumor recurrence or metastasis, and CA19-9 had returned to the normal range. The patient reported that her daily life had returned to normal and that she was satisfied with the treatment. We did not observe any adverse or unexpected events related to the surgery. Although no recurrence or metastasis was observed during the 24-month follow-up period, longer follow-up is still needed because MCN-L has malignant potential and late recurrence cannot be completely excluded.

## 9. Discussion

### 9.1. Pathogenesis and etiology

The pathogenesis of MCN-L remains incompletely understood. One proposed explanation is that it may arise from ectopic ovarian-like mesenchymal tissue or from developmental abnormalities of the embryonic foregut, which may explain why similar mucinous cystic tumors have also been described in the gastrointestinal tract, biliary tract, and urinary system.^[1]^

Another widely discussed hypothesis is that hormonal factors may contribute to tumor development and growth. This view is mainly based on the marked female predominance of MCN-L and the characteristic presence of ovarian-type stroma beneath the epithelial lining. In addition, expression of estrogen receptor and progesterone receptor has been demonstrated in the stromal component of these tumors, including in our case, which supports a possible hormone-responsive biological behavior.^[2–4]^ Although a direct causal relationship has not been fully established, these findings suggest that estrogen-related stimulation may promote cyst enlargement and epithelial proliferation in at least some patients. For this reason, the hormonal background of MCN-L deserves more attention when interpreting both its pathologic features and its clinical behavior.

### 9.2. Clinical manifestations and auxiliary examinations

The clinical symptoms of MCN-L are often nonspecific. When the lesion compresses the biliary system, patients may have upper abdominal discomfort, abdominal distension, or jaundice. Some patients present with abdominal pain, nausea, vomiting, fever, or a palpable abdominal mass, and these symptoms may be aggravated by changes in body position.^[2,3]^ However, there are also patients like the one in our report who have almost no discomfort. Previous studies indicate that completely asymptomatic patients account for < 15% of all cases.^[4]^ Our patient belonged to this small group, and the lesion was detected only during routine screening.

The diagnostic criteria for MCN-L mainly include the presence of ovarian-like stroma in the cyst wall and mucin-secreting epithelium on histological examination.^[5]^ Measuring CA19-9 in cystic fluid may also be helpful. A markedly elevated CA19-9 level in cyst fluid has been reported in many cases, and can serve as an auxiliary diagnostic indicator. In our case, the serum CA19-9 level was obviously elevated (553 IU/mL), which is consistent with previous reports suggesting that CA19-9 is a useful marker for MCN-L.^[6]^ Nevertheless, in daily practice, clinicians usually rely on imaging features and intraoperative findings before pathology is available.

In this case, the main diagnostic difficulty was that the lesion initially appeared similar to a benign hepatic cystic lesion, particularly because the patient had no obvious symptoms. On initial ultrasound, it was recognized simply as a hepatic cystic lesion. However, its giant size, multilocular architecture, visible septa, and mild enhancement of the cyst wall and septa on contrast-enhanced imaging argued against a simple liver cyst. Hydatid disease was also taken into consideration during differential diagnosis, but the absence of daughter cysts, calcified rim, detached membranes, epidemiologic history, and serologic evidence made this possibility less likely. Therefore, although imaging alone could not establish a definitive diagnosis, the overall findings raised our suspicion for MCN-L before surgery.

On ultrasound, MCN-L often appears as a single, large multilocular cystic mass or, less commonly, as scattered unilocular cysts. The cyst wall may have uneven thickness, and internal echogenic septa may be seen. Color Doppler sometimes shows blood flow within the septa.^[7]^ On CT, MCN-L typically presents as a large, well-circumscribed multilocular cystic lesion with clear boundaries, low attenuation contents, and variable wall thickness. Septa and mural nodules may enhance after contrast injection.

MRI can show the capsule, septa, and mural nodules more clearly than CT. Septal or mural enhancement after contrast administration is considered a sensitive imaging sign of MCN-L.^[8–10]^ On T2-weighted images, the fluid within the cysts usually appears as high signal intensity, whereas areas containing calcification or blood products may show low signal intensity margins.

Magnetic resonance cholangiopancreatography is helpful to assess whether cystic lesions communicate with the biliary system and to differentiate MCN-L from other cystic liver diseases.^[11]^ When the diagnosis remains unclear after imaging, frozen biopsy of the cyst wall during surgery is also an accurate way to confirm MCN-L.^[12]^

### 9.3. Differential diagnosis

#### 9.3.1. Simple liver cyst

A simple liver cyst usually has a thin and smooth wall, homogeneous fluid content, and no internal septa or mural nodules. Enhancement is generally absent after contrast administration. In our case, the lesion was multilocular and showed septal enhancement, which made a simple cyst less likely.

#### 9.3.2. Liver hydatidosis

Hydatid disease may also present as a cystic liver lesion, but it more often shows daughter cysts, internal membranes, rim calcification, or other characteristic signs such as the “water lily sign.” In addition, epidemiologic exposure history and positive serologic tests may support that diagnosis. None of these findings were present in our patient, so hydatid disease was considered but not favored.

#### 9.3.3. Liver hamartoma

Mesenchymal hamartoma is seen mainly in infants and young children rather than in adults. Although it may appear as a multilocular cystic lesion, the age at presentation and imaging pattern are usually different from those of MCN-L. In our patient, the adult age, elevated CA19-9 level, multilocular cystic morphology, and final histopathologic findings all supported MCN-L over liver hamartoma.^[13]^

### 9.4. Treatment and prognosis

Treatment options for MCN-L include percutaneous aspiration, surgical fenestration or marsupialization, and surgical resection. Because MCN-L has malignant potential and simple drainage often leads to recurrence, most authors believe that complete surgical removal is the best treatment.^[3,4]^ Surgical techniques mainly include traditional open liver resection and laparoscopic liver resection.

In recent years, laparoscopic liver resection has progressed rapidly. For suitable patients, laparoscopic surgery for mucinous cystic liver tumors can achieve similar oncologic results to open surgery, with advantages such as less blood loss, smaller wounds, and faster recovery.^[14]^ Compared with open hepatectomy, laparoscopic hepatectomy has been reported to have a lower risk of major morbidity and shorter hospital stay.^[15]^ Other studies also highlight reduced postoperative pain, better cosmetic outcomes, and lower mortality with laparoscopic approaches.^[16]^

In our case, the tumor measured about 20 cm, occupied most of segment IV, and was closely related to the middle hepatic vein and left hepatic vein. After multidisciplinary discussion, we believed that open resection would allow better exposure, safer vascular control, and a lower risk of intraoperative rupture. This was particularly important because en bloc removal with negative margins was one of our main operative goals. Therefore, although laparoscopic hepatectomy is an attractive option in selected cases, open segment IV hepatectomy was a more prudent and reliable choice for this patient.

After surgery, patients may develop complications such as bile leakage, intra-abdominal infection, bleeding, pleural effusion, or liver dysfunction. Careful surgical technique, thorough hemostasis, and proper drainage can reduce these complications. Postoperatively, close monitoring and timely rehydration, liver protection, and infection prevention are also important for recovery.^[9,17,18]^

## 10. Conclusion

MCN-L is a rare mucinous cystic tumor of the liver that occurs predominantly in women, and its pathogenesis has not yet been fully clarified.^[8,9]^ Because the clinical presentation is often subtle or even absent, preoperative diagnosis may be challenging, particularly when the lesion is initially regarded as a benign hepatic cyst. Contrast-enhanced imaging, especially MRI, is valuable for identifying multilocular structure, septa, and cyst wall enhancement, but the final diagnosis still depends on histopathology and the demonstration of ovarian-type stroma.^[10]^

Treatment should be individualized according to tumor size, location, internal characteristics, and relationship to major vascular structures. Complete surgical excision with negative margins remains the key to preventing recurrence and malignant transformation. In our patient, open hepatectomy was chosen because the tumor was giant and centrally located, making safe laparoscopic resection less suitable. Although the postoperative course was uneventful and no recurrence was observed during 24 months of follow-up, continued surveillance is still warranted.^[7]^

This case reminds us that when a large hepatic cystic lesion is incidentally found in an asymptomatic young woman, especially in the presence of elevated CA19-9 and septal enhancement on imaging, MCN-L should remain in the differential diagnosis.

## Acknowledgments

We sincerely thank the patient and her family for their trust and for providing consent for the publication of this case.

## Author contributions

**Funding acquisition:** Jin Wang.

**Resources:** Jin Wang.

**Writing – review & editing:** Hao-nan Cheng, Ya-kang Jia, Meng-wei Cai, Jun-yan Xu, Wei Guo, Jin Wang.
